# Importance of thorough tissue and cellular level characterization of targeted drugs in the evaluation of pharmacodynamic effects

**DOI:** 10.1371/journal.pone.0224917

**Published:** 2019-11-14

**Authors:** Dustin K. Bauknight, Victoria Osinski, Siva Sai Krishna Dasa, Anh T. Nguyen, Melissa A. Marshall, Julia Hartman, Matthew Harms, Gavin O’Mahony, Jeremie Boucher, Alexander L. Klibanov, Coleen A. McNamara, Kimberly A. Kelly

**Affiliations:** 1 Department of Biomedical Engineering, University of Virginia, Charlottesville, VA, United States of America; 2 Cancer Center, University of Virginia, Charlottesville, VA, United States of America; 3 Robert M. Berne Cardiovascular Research Center, University of Virginia, Charlottesville, VA, United States of America; 4 Department of Pathology, University of Virginia, Charlottesville, VA, United States of America; 5 Research and Early Development, Cardiovascular, Renal and Metabolism (CVRM), BioPharmaceuticals R&D, AstraZeneca, Gothenburg, Sweden; 6 The Lundberg Laboratory for Diabetes Research, University of Gothenburg, Gothenburg, Sweden; 7 Wallenberg Centre for Molecular and Translational Medicine, University of Gothenburg, Gothenburg, Sweden; 8 Department of Medicine, Division of Cardiovascular Medicine, University of Virginia, Charlottesville, VA, United States of America; Chapman University, UNITED STATES

## Abstract

Targeted nanoparticle delivery is a promising strategy for increasing efficacy and limiting side effects of therapeutics. When designing a targeted liposomal formulation, the *in vivo* biodistribution of the particles must be characterized to determine the value of the targeting approach. Peroxisome proliferator-activated receptor (PPAR) agonists effectively treat metabolic syndrome by decreasing dyslipidemia and insulin resistance but side effects have limited their use, making them a class of compounds that could benefit from targeted liposomal delivery. The adipose targeting sequence peptide (ATS) could fit this role, as it has been shown to bind to adipose tissue endothelium and induce weight loss when delivered conjugated to a pro-apoptotic peptide. To date, however, a full assessment of ATS *in vivo* biodistribution has not been reported, leaving important unanswered questions regarding the exact mechanisms whereby ATS targeting enhances therapeutic efficacy. We designed this study to evaluate the biodistribution of ATS-conjugated liposomes loaded with the PPARα/γ dual agonist tesaglitazar in leptin-deficient *ob/ob* mice. The ATS-liposome biodistribution in adipose tissue and other organs was examined at the cellular and tissue level using microscopy, flow cytometry, and fluorescent molecular tomography. Changes in metabolic parameters and gene expression were measured by target and off-target tissue responses to the treatment. Unexpectedly, ATS targeting did not increase liposomal uptake in adipose relative to other tissues, but did increase uptake in the kidneys. Targeting also did not significantly alter metabolic parameters. Analysis of the liposome cellular distribution in the stromal vascular fraction with flow cytometry revealed high uptake by multiple cell types. Our findings highlight the need for thorough study of *in vivo* biodistribution when evaluating a targeted therapy.

## Introduction

Several successful liposomal formulations have received FDA approval, including Onivyde in 2015 [1–4]. With superior safety profiles and increased efficacy, liposomes have demonstrated clinically proven effectiveness as a drug delivery vehicle. Liposomes are used to encapsulate a drug and alter its pharmacokinetics (PK) and pharmacodynamics. This approach is broadly useful because liposomes can store water-soluble compounds in their core and hydrophobic compounds in the lipid bilayer. The clinical success of liposomes and their potential to increase a drug’s therapeutic index makes them an attractive option for drug delivery. Targeting molecules such as antibodies and peptides can be conjugated to lipids on the liposome’s surface, potentially enhancing efficacy by altering the biodistribution to increase uptake in target tissues or cells. Peptide-targeted liposomes are a promising approach to deliver drugs to specific cell types and, with their ease of synthesis and cost effectiveness, face fewer regulatory hurdles than antibody-targeted liposomes, which would be classified as biologics.

Tesaglitazar is part of a larger family of PPARα/γ dual agonists, which effectively improve insulin sensitivity and dyslipidemia in patients with diabetes and obesity-associated dysmetabolism. In fact, tesaglitazar effectively improves insulin sensitivity and lowers circulating lipid levels better than PPARγ-selective agonists in subjects with metabolic syndrome [5–7]. It is also effective at treating symptoms of dysmetabolism in rodent models of obesity and diabetes [8, 9]. PPARγ agonists are effective at increasing insulin sensitivity by directing and storing lipids safely into adipose tissues. PPARα agonists can counteract the lipogenic effect of PPARγ agonism via increased fatty acid β-oxidation in the liver, thus making PPARα/γ dual agonism an appealing therapeutic option [10, 11]. However, PPARγ and -α agonists have been shown to cause a number of side effects in patients including edema, weight gain and heart failure [12, 13]. Specifically, tesaglitazar’s clinical relevance was limited by off-target effects in the kidneys: increasing glomerular filtration rate (GFR) and serum creatinine levels [5–7, 13–15]. Tesaglitazar stood out as a good candidate compound for this targeting study as it has demonstrated greater therapeutic efficacy in populations with dysmetabolism and because it has known biomarkers of drug action in adipose, kidney and liver tissues that can serve as readouts for tissue-specific drug action, which are complementary to image-based biodistribution assays. Furthermore, liposomal delivery enhances uptake by the reticuloendothelial system including the liver and spleen reducing drug exposure in the kidneys [16] and thus potentially reducing unwanted side effects of tesaglitazar.

The Adipose Targeting Sequence (ATS) was discovered by Kolonin *et al*. in 2004 with an *in vivo* phage display screen on mouse adipose tissue vasculature [17]. Immunohistochemical staining from the study suggested enhanced binding to adipose tissue by a specific interaction with the protein prohibitin [17]. Hossen *et al*. demonstrated that ATS-targeted liposomes undergo prohibitin-dependent uptake by adipose endothelial cells (EC) *in vitro* [18]. Groups have also utilized ATS to develop multiple targeted delivery systems including nanoparticles for delivery of a pro-apoptotic peptide [18, 19] or PPARγ agonist rosiglitazone [20], oligopeptide complexes for gene therapy [21], and ATS conjugated to the pro-apoptotide peptide KLAKKLAK [17, 22, 23] (Table 1). Many of these studies demonstrate enhanced treatment efficacy over controls, suggesting ATS-targeting of tesaglitazar may be an effective delivery system. However, none of these studies quantified *in vivo* biodistribution of ATS (Table 1). Given the role that biodistribution studies play in ensuring or determining translation of drug delivery systems to the clinic especially for understanding the potential for on target and off target toxicities [16], conducting a thorough evaluation of ATS-targeted biodistribution is important and necessary.

**Table 1:** Adipose targeting sequence publication summary.

Publication year and citation number	Disease model	Drug delivery system	Controls used	Treatment effects	ATS-specificity and biodistribution assays and quantification	Other observations
2004 [17]	Obesity, Mouse	Pro-apoptotic peptide (KLAKLAK)_2_ conjugated to ATS	Equimolar mixture of the ATS peptide and untargeted (KLAKLAK)_2_ peptide	Reduced overall weight gain, lowered circulating lipids and leptin levels	Immunohistochemistry: Co-localization with isolectin in WAT 5 minutes post-IV injection No quantification	Demonstrated expression of prohibitin in adipose tissue
2010	N/A, Primary cultured AT-derived ECs	Fluorescently-labeled, ATS-conjugated liposomes	Scrambled peptide-conjugated liposomes	N/A	Confocal laser scanning microscopy: co-localization of ECs with liposomes No quantification	Pre-treatment with a prohibitin-specific antibody blocked liposome uptake
2010	Obesity, Mouse and Rat	Pro-apoptotic peptide (KLAKLAK)_2_ conjugated to ATS	Vehicle, Control peptide (CKGGRAKDC)	Attenuated weight gain, reduced energy intake, reduced adipose and circulating leptin levels	None assessed	Reduced expression level of POMC in the hypothalamus with treatment
2011	Obesity, Non-human primate	Pro-apoptotic peptide (KLAKLAK)_2_ conjugated to ATS, “adipotide”	Saline	Weight loss, improved insulin tolerance, increased creatinine levels, mild kidney tubular degeneration	None assessed	BUN levels spiked in treated macaques around day 8 of treatment, but were not significantly different for the remainder of the treatment
2012	Obesity, Mouse	Fluorescently-labeled, ATS-conjugated liposomes containing pro-apoptotic peptide (KLAKLAK)_2_	No peptide-conjugated liposomes containing pro-apoptotic peptide	Attenuated weight gain, decreased adipose tissue vascular density	Confocal laser scanning miscroscopy: Liposome and isolectin co-localization in in SC AT and liver Liposome fluorescence normalized to isolectin fluorescence was quantified	Increased uptake of untargeted liposomes into adipose tissue in obese mice compared to lean mice Note: Images and quantification of liver and SC AT were conducted only with peptide-targeted liposomes
2014	Obesity, Mouse + Cultured adipocytes	shFABP4-ATS peptide complex	shLuciferase-ATS peptide complex, Naked shFABP4, no oligopeptide complex	Reduced weight gain, improved glucose and insulin tolerance	Probe-type confocal endomicroscopy: co-localization with isolectin in adipose, liver, and kidney immediately following IV injection No quantification	ATS-colocalized with prohibitin on the plasma membrane of adipocytes
2016 [20]	Obesity, Mouse	Fluorescently-labeled, rosiglitazone-loaded nanoparticles with ATS peptide	Rosiglitazone-loaded nanoparticles without peptide, unencapsulated rosiglitazone, no treatment	Increased adipose vascular density, reduced weight gain*, reduced circulating insulin and lipids levels (compared to no treatment)	IVIS: ex vivo tissues (Epid AT, SC AT, Liver) 12 hours post-IV injection No quantification	Untargeted liposomes also reduced circulating insulin and lipid levels Note: Weight gain for all experimental groups was not plotted on the same graph

Additionally, while *in vivo* and *in vitro* prohibitin expression on endothelium has been published, it is also known that prohibitin is expressed on many other cell types including intestinal epithelial cells, adipocytes, and immune cells [24–27], leaving us to question whether the mechanisms by which ATS induces therapeutic efficacy is through targeting to adipose tissue vasculature, or by other means. Furthermore, Xue *et al*. published their findings suggesting that ATS-targeted delivery of rosiglitazone in ATS-targeted nanoparticles improves metabolic parameters, such as fasting insulin, in obese mice to a greater extent than unencapsulated rosiglitazone and untargeted nanoparticles [20]. Interestingly, data from this study also suggests that untargeted nanoparticles improved metabolic parameters over unencapsulated rosiglitazone. This finding coupled with the finding that obese adipose tissue takes up substantially more liposomes than lean adipose tissue [19] suggests that untargeted liposomes may be an alternative delivery system sufficient to improve symptoms of dysmetabolism in obese mice and potentially humans.

Therefore, we developed ATS-targeted, tesaglitazar-loaded, fluorescently labeled, liposomes to undertake a thorough evaluation of their targeting efficiency and examine the efficacy of both targeted and untargeted tesaglitazar-loaded liposomes in improving metabolic parameters. Importantly, we undertook the first fulsome study of the biodistribution of ATS-liposome targeting including quantification of whole tissue uptake rather than via limited sampling and quantification of tissue uptake using microscopy. We assessed ATS-liposome uptake on the cellular and tissue level using flow cytometry, *ex vivo* fluorescence molecular tomography (FMT), and microscopy. The use of fluorescent liposomes enables measurements from all three of these methods from a single animal. Ex vivo tomographic imaging such as ex vivo FMT is an important part of this approach because it can quantitatively assess the biodistribution (g,16). Metabolic assays, and RT-qPCR were utilized to assess the effects of liposomal delivery of tesaglitazar on metabolism and PPARα/γ agonism in multiple tissues. With these experiments, we sought to elucidate the cell types and tissues to which the ATS peptide on liposomes is targeted and validate whether ATS targeting attenuates liposomal uptake in off-target tissues such as the kidney.

In summary, we found that ATS-targeted liposomes did not significantly improve metabolic outcomes over untargeted liposomes and they did not demonstrate significant increases in uptake in the adipose vasculature or adipose tissue. Interestingly, we observed that while there was binding to adipocytes and endothelium in the adipose depots, the majority of the ATS-peptide and non-targeted liposomes accumulate in macrophages, demonstrating the need for a thorough validation of cell binding profiles for targeting experiments.

## Methods

### Animals

Male C57Bl/6 leptin-deficient (*ob/ob*) mice were purchased from Jackson Labs (Stock # 000632). All animal experiments performed in this study were approved by the Institutional Animal Care and Use Committee of the University of Virginia.

### Metabolic studies

Mice were fasted for approximately 4 hours in wood chip-lined cages with water provided *ad libitum*. Following fasting, a small tail snip was made to obtain blood for measuring blood glucose levels with a glucometer (OneTouch Ultra 2 glucometer and UniStrip Technologies 24850). Mice were then placed under anesthesia (isofluorane) and blood was collected via retro-orbital bleed. Blood was treated with EDTA (0.5 M) and spun down to collect plasma to measure insulin (ALPCO, 80-INSMR-CH01) and triglyceride levels (Sigma TR0100).

### Tissue harvest and processing

In general, mice were euthanized by CO_2_ overdose. Mice were perfused through the left ventricle (after cutting the right atrium) with 10 mL PBS supplemented with 0.5 mM EDTA followed by 5–10 mL of PBS before harvesting all other tissues. Inguinal lymph nodes were removed before harvesting the inguinal (subcutaneous) adipose tissue. All tissues harvested for RNA extraction were flash frozen in liquid nitrogen and stored at -80°C.

#### Bone marrow cells

Following perfusion, rear femurs and tibias were harvested and excess muscle and tissue removed. The ends of each bone were cut away to access the marrow. Using 5 mL of PBS per bone, each bone was flushed using a syringe. Cell suspensions were spun and treated with AKC lysis buffer to lyse remaining red blood cells. Cells were then washed with FACS buffer (PBS, 0.05% NaN_3_, 1% BSA) to be stained for flow cytometry.

#### Adipose stromal vascular fraction (SVF) cells

Whole adipose tissue was placed in digestion buffer (0.12M NaCl, 4.7mM KCl, 1.3 mM CaCl_2_.2H_2_O, 1.2 mM KH_2_PO_4_, 1.2 mM MgSO_4_7H_2_O, 40 mM HEPES (pH 7.5), 2.5% BSA, 200 nM adenosine, 50 U/μL Collagenase Type 1), minced, and incubated at 37°C, shaking, for ~45 minutes. To reduce the possibility of disproportionate cell yields and altered cell surface markers in the SVF, we utilized digestion methods that have been established for flow cytometric analysis of cell populations and utilized by many. Methods employing collagenase-based digestion of adipose tissue have been used to not only assess immune cell populations, but also isolate progenitor cell populations and vascular cell populations including endothelial cells [28–30]. Digested tissue was then washed with FACS buffer, and pelleted. Cells were treated with AKC lysis buffer to lyse remaining red blood cells and then filtered through a 70 μm filter to remove undigested tissue and/or matrix proteins. Cells were then stained for flow cytometry or pelleted and flash frozen for RNA extraction. The panel of proteins probed for in this study have not demonstrated reduced expression as a result of digestion. Furthermore, while processing tissues, samples are kept on ice whenever possible to reduce cell surface changes that might also alter our results.

#### Liver and kidney

Following perfusion, whole livers and kidneys were harvested. To prepare tissue for RNA extraction, whole tissue or tissue aliquots was homogenized in Trizol. To prepare for immunofluorescence staining, tissues were fixed overnight in 4% PFA at 4°C.

### Preparation and characterization of liposomes

Liposomes were initially prepared with the remote loading attractant calcium acetate using the reverse-phase evaporation technique [31] with DSPC, cholesterol and PEG-2000 DSPE at a mass ratio of 2:1:1 (phospholipids were from Avanti or Lipoid; cholesterol from Sigma). Additionally, DiD lipid dye (1 mg/mL) was added during this step to fluorescently label the liposomes. Lipid dyes like DiO, DiD and DiI are routinely used for liposome research; they are considered non-exchangeable [32].

An ether-chloroform solution of lipids and dye was mixed with aqueous calcium acetate (1 M, pH 7.4). The ratio between organic and aqueous phase was 4:1. The mixture was subjected to emulsification by sonication (XL2020, Misonix, 50% power, 30 sec) and then organic solvents were removed under vacuum using a rotary evaporator (Re111, Buchi) connected to a vacuum line. Nuclepore filtration resulted in ~120 nm particles. External Ca-acetate was removed by Zeba spin-column and to half of the batch, aqueous tesaglitazar at 3.3 mg/ml in HEPES buffer (pH 7.4) was added and incubated with mixing at 37°C for 1 hour. External unentrapped tesaglitazar was removed from liposomes with a Zeba spin-column. Tesaglitazar concentration was quantified using UV absorbance at 270nm in order to calculate the drug-to-lipid ratio.

The ATS peptide (NH_2_-GKGGRAKDGGSC) was synthesized by the Tufts University peptide synthesis core facility using standard FMOC chemistry and Rink-Amide resin (Tufts University, Boston, MA). It was conjugated to DSPE-PEG_3400_-maleimide via the C-terminal cysteine as follows: DSPE-PEG_3400_-maleimide (9.5 mg) was first dissolved in 200 μL of methanol. PBS / 0.5 mM EDTA (800 μL) was then added to prepare the aqueous micellar solution. The ATS peptide was immediately dissolved in the micellar solution of DSPE-PEG_3400_-maleimide, under argon. The reaction mixture was left overnight at 4°C followed by dialysis in PBS (1 × 2 L) followed by dialysis in water (2 × 2 L), to remove free peptide and salts from the conjugated micelles. The purified peptide-PEG-DSPE was then lyophilized and this lipid powder was used in liposomal preparations. Peptide lipid conjugates were added to the liposomes after loading, using a post-insertion method. Peptide micelles were formed by hydrating the peptide-lipid conjugate in a buffered solution. The micelles were mixed with the tesaglitazar liposomes for 1 hour at 60°C. The resulting liposomes were characterized by Nanoparticle Tracking Analysis (NTA) (Nanosight NS300, Malvern Instruments Ltd., Worcestershire, UK) to determine particle size and concentration. Zeta potentials of the liposomal formulations were determined in 1 mM HEPES buffer pH 7.4 at a dilution of 1:100 using zeta potential measurements (Zetasizer 3000; Malvern Instruments, Worcestershire, UK). Liposomes were also imaged using cryoTEM to assess particle structure.

### *In vivo* liposome treatments

Liposomes were injected in mice (n = 6) via the tail vein at a dose of 1 μmol of tesaglitazar/kg/day. Timing of injections is specified in figures where appropriate.

### *Ex vivo* biodistribution

Liposome uptake was measured using *ex vivo* FMT imaging of organs to determine the amount of DiD present in tissues and was represented as percentage of injected dose per gram of tissue (%ID / g). %ID / g was calculated using the following equation: %ID / g = (Tissue Value * 100) / (Total injected dose) where the total injected dose was the sum of injected doses from each of the three days of injections. Organs were imaged using the 680nm laser of the FMT 4000 system (PerkinElmer, Waltham, MA).

### Flow cytometry

All cells were stained with Live/Dead (Fisher) in PBS for 30 minutes at 4°C then washed with FACS buffer. Next, the cells were stained with fluorophore-conjugated antibodies against cell surface proteins (Table 2) in FACS buffer or Brilliant Violet Stain Buffer (BD, if more than one Brilliant Violet fluorophore was used at one time) for 25 minutes at 4°C then washed with FACS buffer. Cells were then fixed with 2% PFA for 7–10 minutes at room temperature and washed with FACS buffer. Finally, cells were resuspended in FACS buffer and stored at 4°C until analyzed. Cells were run on the Attune and compensated and analyzed in FlowJo Version 9.

**Table 2:** Antibody list.

Marker	Fluorochrome(s)	Company	Clone
CD31	FITC	eBioscience	390
CD11b	BV421, FITC	BioLegend	M1/70
CD19	PE	eBiosciences	eBio1D3
CD45	PE-CF495	BD	30-F11
F4/80	PE-Cy7	BioLegend	BM8
CD3	FITC	Pharmingen	145-2C11

### Real-time polymerase chain reaction

RNA was extracted from tissues and cells using Trizol extraction. 1 μg of RNA was then treated with DNase (Invitrogen) and used to reverse transcribe cDNA using an iScript cDNA synthesis kit (BioRad). To quantify gene expression, cDNA was diluted 1:10 in water and combined with 0.5mM forward and reverse primers (Table 3) and SYBR Green (SensiFast, BioLine). Semi-quantitative real-time PCR was performed on a CFX96 Real-Time System with an annealing temperature of 60°C for all reactions (BioRad). Data were calculated by the ΔΔCt method and expressed in arbitrary units that were normalized to *18s* or *Cyclophilin* levels.

**Table 3:** Primer sequences.

Gene	Forward (5’-3’)	Reverse (5’-3’)
*18s*	CGGCTACCACATCCAAGGAA	AGCTGGAATTACCGCGGC
*Cyclophilin*	TGCCGGAGTCGACAATGAT	TGGAGAGCACCAAGACAGACA
*Ehhadh*	TCGAATGTTGGCTCCCTATTAC	CCAGCTTCACAGAGCATATCA
*Fabp3*	AGGCAGCATGGTGCTGAGCTG	AGGCAGCATGGTGCTGAGCTG
*Nos2*	CGAAACGCTTCACTTCCAA	TGAGCCTATATTGCTGTGGCT
*Serpine-1*	GTAAACGAGAGCGGCACA	CGAACCACAAAGAGAAAGGA
*Pdk4*	AATTTCCAGGCCAACCAATCC	GGTCAAGGAAGGACGGTTTTC
*Tgfb1*	AGCCCGAAGCGGACTACTAT	CTGTGTGAGATGTCTTTGGTTTTC

### Immunofluorescence

Livers and kidneys were fixed in 4% PFA and then subjected to a sucrose gradient (10% O/N, 20% 6 hrs, 30% O/N) at 4°C, rotating. Then, tissues were embedded in OCT and 10 μm sections obtained. For staining, tissue sections were permeabilized with 0.25% Triton-100 in PBS, and then washed in PBS. Sections were blocked with 0.6% fish skin gelatin with 10% serum in PBS, then incubated with rat anti-CLECSF13 antibody (R&D Systems, MAB2784) at a 1:250 dilution in 0.6% fish skin gelatin with 10% serum in PBS overnight at 4°C. Sections were washed as before and then incubated with donkey anti-rat Dylight 550 secondary antibody at a 1:250 dilution and mouse anti-Acta2-FITC (Sigma, F3777) at a 1:500 dilution. Following one final wash, slides were counterstained with DAPI and coverslipped using ProLong Gold (Life Technologies). Z-stack images were obtained using Zeiss LSM700 confocal microscope, 20X objective. Figures shown are maximal intensity projection images.

### Whole-mounted imaging

Aliquots of epididymal and subcutaneous adipose were fixed in 4% PFA then washed in PBS. Adipose was blocked and permeabilized in 5% BSA, 0.3% Triton in PBS before incubating overnight with CD68-PE conjugate (Biolegend, Clone FA-11) and Isolectin GS-IB_4_ AF488 conjugate (Thermofisher) or CD31 AF488 (Biolegend, Clone MEC13.3) at 4°C. After a final wash, samples were mounted in a 1:1 solution of PBS: Glycerol and digital images were acquired using confocal microscopy (Nikon Instruments Incorporated, Model TE200-E2; 20X objective). Images were processed using ImageJ software.

### Statistics

All statistical analysis was performed using Prism 7 (GraphPad Software, Inc.). Because sample n < 15 for all experiments, normal distribution could not be determined. Mann-Whitney tests were used to compare two experimental groups, Kruskal-Wallis tests were used to compare three experimental groups. Data are generally expressed as mean ± SD. P values are specified in figure legends.

## Results

### Liposome preparation with tesaglitazar remote loading and post-insertion ATS targeting

**Fig 1:**
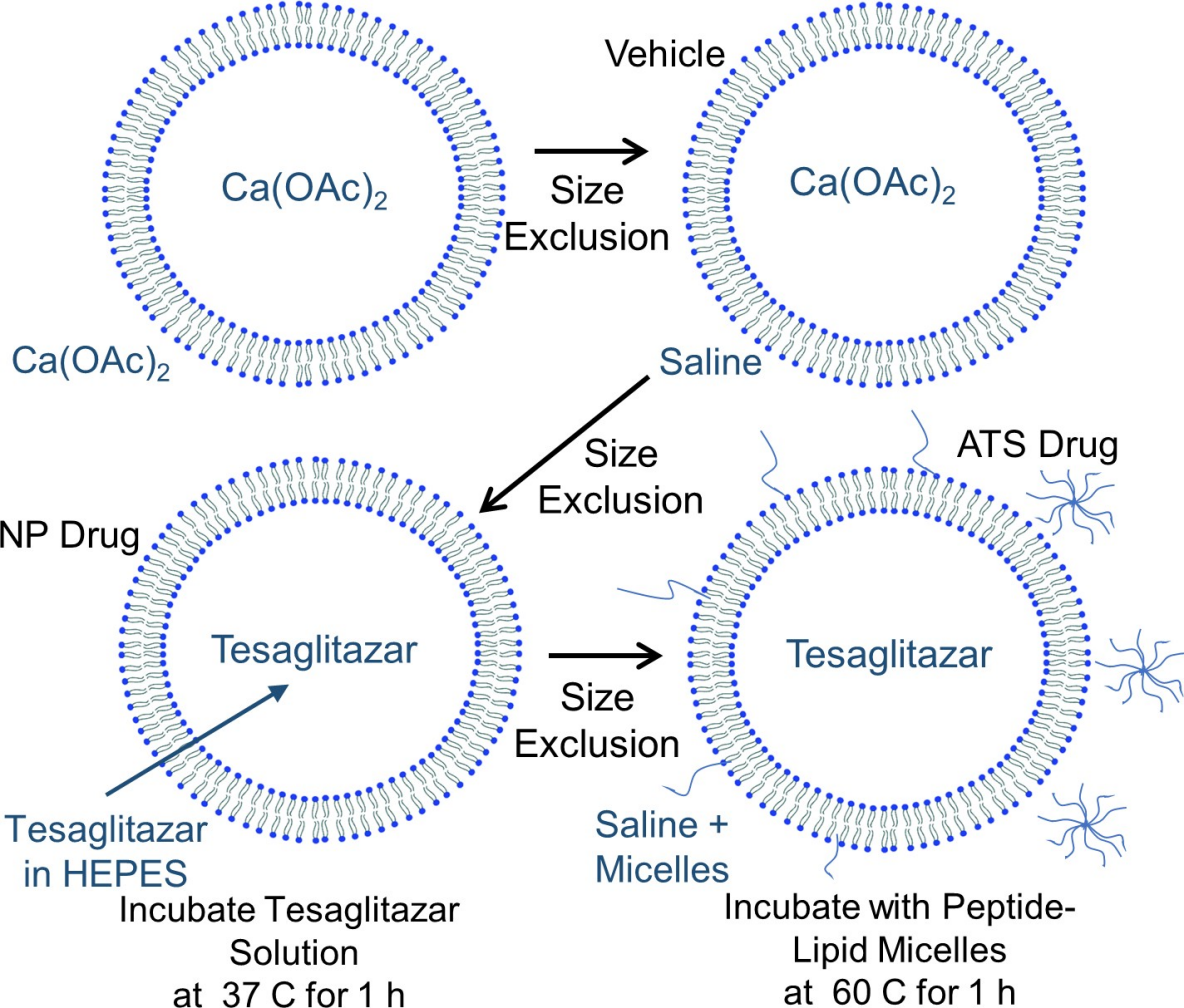
Targeted liposome synthesis. Liposomes were synthesized with reverse phase evaporation, loading them with a 1 M Calcium Acetate Ca(OAc)_2_ solution in preparation for remote loading with PPAR agonist tesaglitazar. Liposome buffer exchanges from Ca(OAc)_2_ to saline, to 3.3 mg/mL tesaglitazar in HEPES, and then back to saline were performed with size exclusion chromatography. Targeting peptides were then added to the liposomes with the post insertion method by incubating liposomes with peptide micelles.

In order to achieve targeted delivery of tesaglitazar to the adipose tissue, we created ATS peptide-conjugated, tesaglitazar-loaded liposomes that were synthesized and characterized by routine methods in our lab [33, 34]. The ATS targeting peptide was chosen because it was previously validated in multiple targeting applications [17, 18, 20, 22]. Liposomes were prepared using reverse phase evaporation with 1 M calcium acetate in the aqueous phase. Tesaglitazar was then loaded into the liposomes by remote loading (Fig 1A) as follows: Tesaglitazar was dissolved in 1 M HEPES and was added to liposomes after removal of exterior calcium acetate by size exclusion chromatography. Loading proceeded for 1 hour at 37°C. Size exclusion chromatography was used to exchange the solution containing unloaded tesaglitazar with saline. HPLC was used to determine the drug-to-lipid ratio, which was 155 μg tesaglitazar/mg lipid. Peptide-lipid conjugates were added to the liposomes using the post insertion method following remote loading (Fig 1). For post insertion, peptide micelles were formed by hydrating the peptide lipid conjugate in PBS. Then the micelles were mixed with the tesaglitazar-loaded liposomes for 1 hour at 60°C. The size and concentration of the liposomes was then characterized by NTA and cryoTEM was used to examine the structure of the liposomes (Table 4 and S1A–S1C Fig). The loaded liposomes were between 100 and 120 nm in diameter for each batch used in this study (S1D Fig), which is directly comparable to previously published ATS-targeted nanoparticles. The zeta potentials were -23.1 mV and -22.0 mV for ATS and non-targeted liposomes, respectively. The liposomes were deliberately designed to replicate the size and surface functionalization of nanoparticles previously used in ATS targeted delivery of PPARγ agonists because these properties are critical to controlling the biodistribution of the particles [35].

**Table 4:** Liposome characterization.

	Batch 1		Batch 2	
	Size (nm)	Concentration (Particles/mL) (1 x 10^12^)	Size (nm)	Concentration (Particles/mL) (1 x 10^12^)
**Vehicle**	**135.1 +/- 3.8**	**25.4 +/- 0.52**	**106.6 +/- 4.8**	**10.3 +/- 0.22**
**NP Drug**	**113.0 +/- 2.5**	**29.9 +/- 0.48**	**101.4 +/- 1.7**	**27.2 +/- 0.76**
**ATS Drug**	**119.4 +/- 0.9**	**23.0 +/- 1.32**	**96.2 +/- 1.5**	**37.8 +/- 1.24**

Liposomes were analyzed with NTA (Nanosight NS300) to determine particle size and concentration

### ATS-targeted tesaglitazar-loaded liposomes do not improve metabolic outcomes over untargeted tesaglitazar liposomes

**Fig 2:**
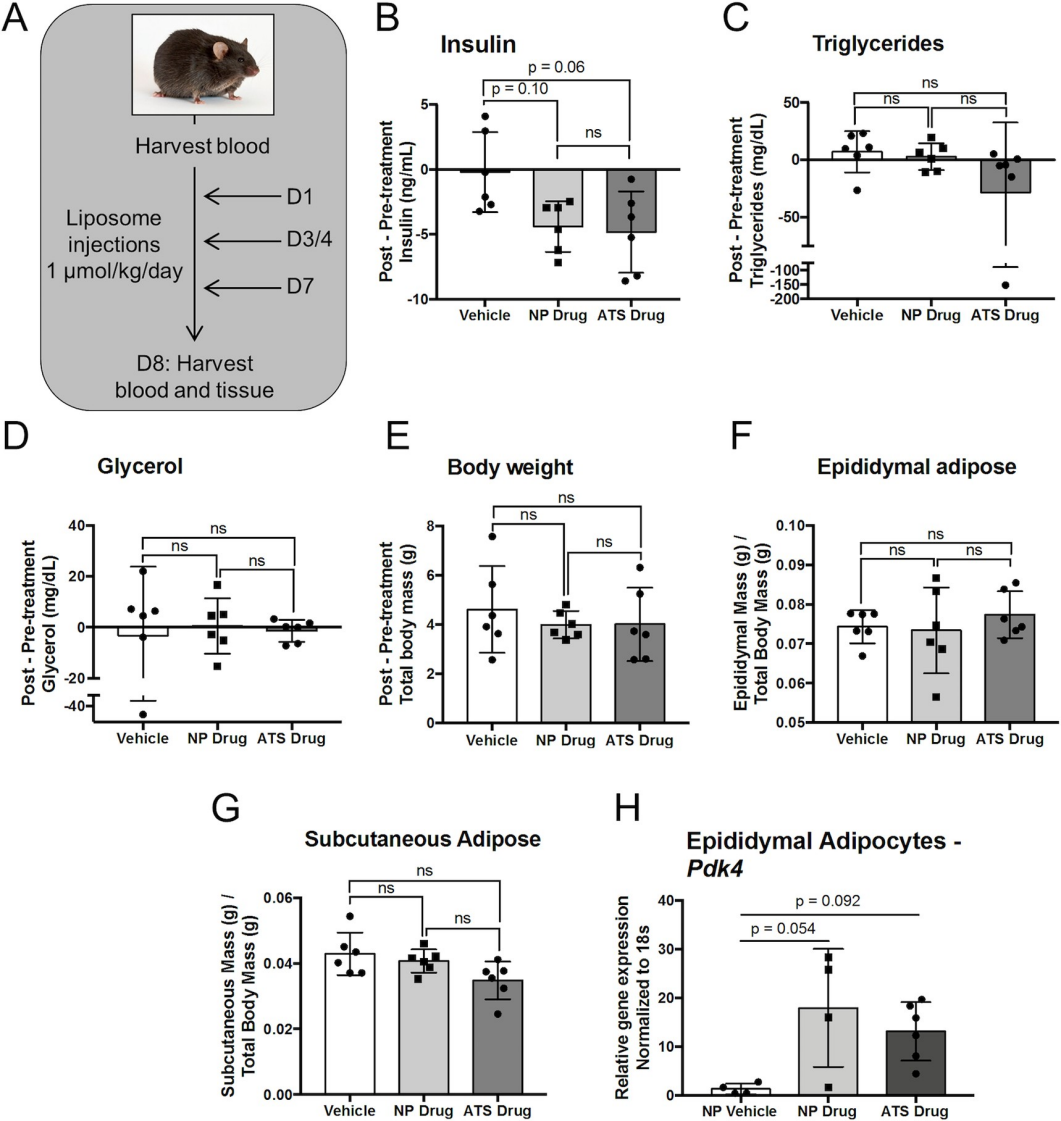
ATS-targeted tesaglitazar-loaded liposomes do not improve metabolic outcomes over untargeted tesaglitazar liposomes. Male *ob/ob* mice were injected three times over the course of one week with liposomes that contained vehicle or tesaglitazar at a concentration of 1μmol of tesaglitazar/kg/day without peptide (NP drug) or with the ATS peptide (ATS drug) (A). Plasma isolated from blood harvested before and after treatment was utilized to measure circulating levels of insulin (B), triglycerides (C), and glycerol (D) and changes from pre-treatment to post-treatment were calculated. The body weight of each animal was also measured before and after treatment and the change in body weight was calculated (E). After treatment, whole epididymal (F) and subcutaneous (G) adipose depots were weighed and tissue weight was normalized to the post-treatment body weight of each mouse. RNA extracted from epididymal adipocytes was utilized to measure relative mRNA expression levels of *Pdk4* (H). *p<0.05, **p<0.01, Kruskal-Wallis test.

To determine if ATS-targeted liposomes could treat dysmetabolism more effectively than untargeted liposomes, we treated mice with targeted (ATS) or untargeted (NP) drug-loaded liposomes three times over the course of one week at increments of two to three days (Fig 2A). Circulating plasma levels of insulin, triglycerides, and glycerol were measured before and after the treatment. Additionally, the expression of the tesaglitazar target gene Pyruvate dehydrogenase kinase 4 (*Pdk4*) in adipocytes was measured after treatment using semi-quantitative real-time PCR. Drug concentration in all liposomes was quantified by UV-vis spectroscopy to ensure that all mice were dosed at 1 μmol/kg/day of tesaglitazar, a dose previously shown to cause beneficial metabolic effects in *ob/ob* mice [9]. Treatment with untargeted drug loaded liposomes (NP drug) and ATS targeted drug loaded liposomes (ATS Drug) both induced a trending reduction in circulating insulin levels compared to vehicle-loaded liposome treatments however, there was not a significant change in circulating insulin between the two drug-loaded treatments (Fig 2B). Neither of the NP drug or ATS drug treatments changed circulating triglyceride (Fig 2C) or glycerol (Fig 2D) levels compared to vehicle-loaded liposome treatments. There was no difference in total body weight gain (Fig 2E), or relative epididymal (Fig 2F) and subcutaneous (Fig 2G) adipose depot mass amongst the three treatment groups. Finally, *Pdk4* mRNA expression in epididymal adipocytes was significantly increased in the NP drug and ATS drug group compared to vehicle controls, but there was no significant difference between NP drug and ATS drug groups (Fig 2H).

### Tesaglitazar-loaded liposomes accumulate in adipose tissue endothelial cells and leukocytes independent of ATS targeting

**Fig 3:**
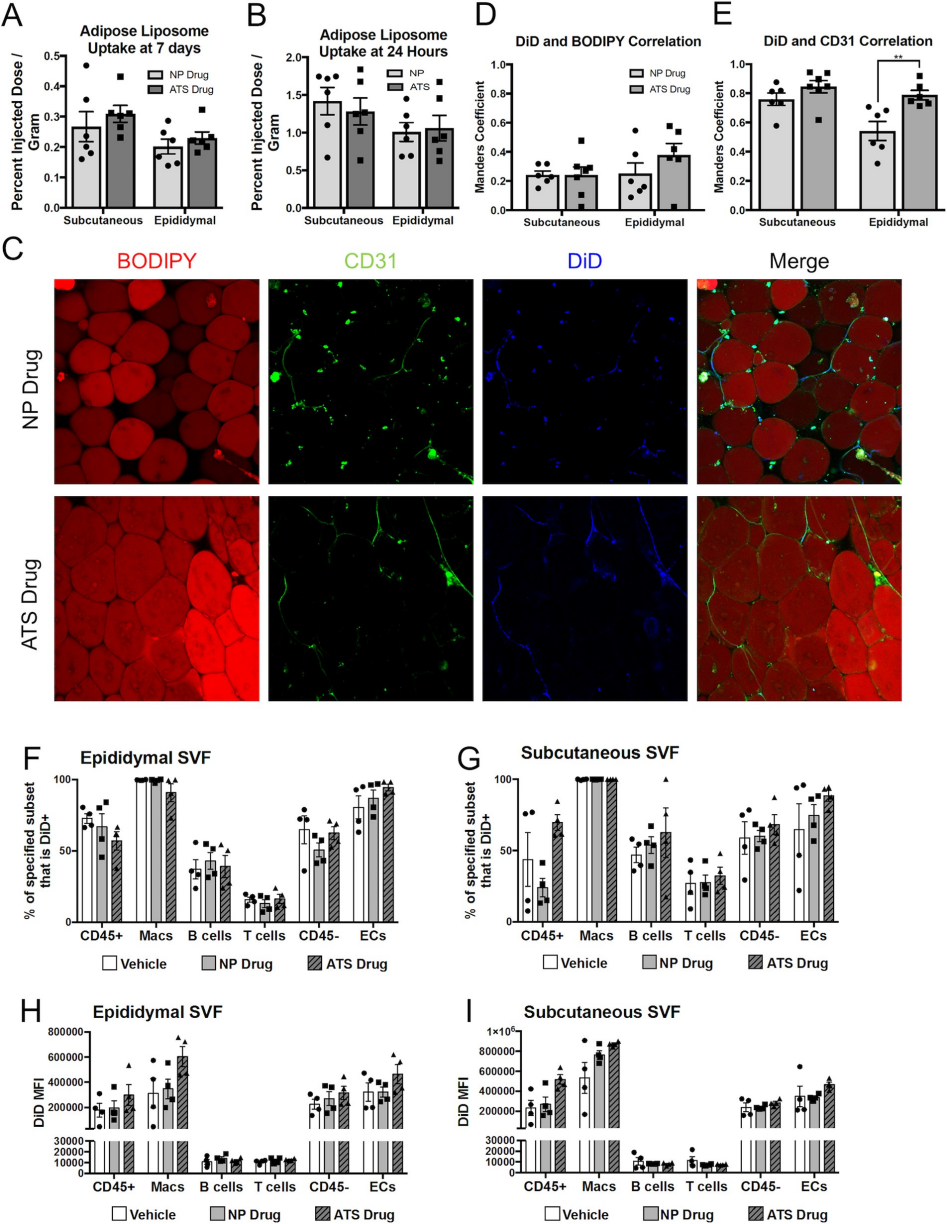
Liposomal uptake is high in macrophages and endothelial cells independent of ATS targeting. *Ob/ob* mice received one or three IV injections of DiD-labeled liposomes and their liposome uptake in adipose tissues was measured one day or one week later, respectively (n = 6). For the one-week study, tesaglitazar-loaded liposomes were used, while the one-day study used vehicle liposomes. At the end of each study, adipose tissues were harvested and *ex vivo* FMT scans were performed to assess liposome uptake (A,B). Whole tissues were also stained with BODIPY and CD31 and were examined with confocal microscopy (C). Manders overlap coefficient was calculated for the liposomes in the epididymal and subcutaneous adipose tissues with six images per comparison (48 total images) (D,E). A one-day flow cytometry study was also performed where the SVF was isolated from adipose depots 24 hours after *ob/ob* mice received IV injections of tesaglitazar loaded liposomes (F-I). **p<0.01, Kruskal-Wallis test.

At the conclusion of the one-week metabolic study of the ATS-targeted and untargeted liposomes, liposomal uptake was measured with *ex vivo* FMT scans of the subcutaneous and epididymal adipose tissues of male *ob/ob* mice. There was not a significant increase in liposomal uptake with ATS-targeted liposomes in either tissue compared to untargeted liposomes (Fig 3A). To further examine ATS-liposome biodistribution, we performed a 24-hour PK study with ATS-targeted and non-targeted vehicle liposomes. *Ex vivo* FMT analysis of adipose depots echoed the one-week metabolic study, with no significant changes seen in either the subcutaneous or epididymal adipose depots (Fig 3B). Cellular uptake of liposomes was examined using confocal microscopy to examine whole tissue samples stained with BODIPY and a CD31 antibody (to stain adipocytes and ECs respectively, representative images, Fig 3C). Mander’s correlation coefficients were calculated for the liposomes in each tissue to determine if ATS targeting increased liposome association with ECs or adipocytes. A significant increase in correlation was seen between epididymal CD31-positive cells and liposomes (Fig 3D and 3E). However, CD31 staining in our samples sometimes localized to crown-like structures that are known to be composed of both ECs and many non-ECs, such as macrophages. Indeed, liposomes did co-localize with both lectin, another established marker of the vasculature, and macrophage marker CD68 in crown-like structures (S2 Fig), creating uncertainty in the determination of liposomal fate that we addressed with multi-marker flow cytometry. We analyzed by flow cytometry the proportion of adipose SVF cells that took up liposomes following a one-week treatment of *ob/ob* mice with tesaglitazar-loaded liposomes (S3 Fig). The majority of ECs and macrophages were liposome positive in the epididymal and subcutaneous adipose depots across all treatment groups, with no significant increases in uptake of ATS-liposomes (Fig 3F and 3G). In addition to assessing the cell types that took up liposomes, DiD mean fluorescence intensities (MFI) were also analyzed to quantify the amount of liposomes taken up per cell. There were no other significant changes between treatment groups (Fig 3H and 3I), but there was a trend towards an increase in total CD45^+^ cells and DiD MFI of CD45^+^ cells between the NP and ATS groups. The lack of increased adipose tissue uptake of ATS-targeted liposomes, coupled with the high uptake in multiple cell types in the adipose tissue, suggests that ATS liposomes are not preferentially taken up by ECs or adipocytes as previously suggested [17, 18, 20]. As macrophages are a cell population affected by PPARα and -γ agonism [36, 37], the presence of such high amounts of liposomes in the macrophage cellular compartment is a confounding factor when analyzing treatment effects.

### Liposomal targeting increases uptake in the kidneys, but does not affect liver and bone marrow uptake

**Fig 4:**
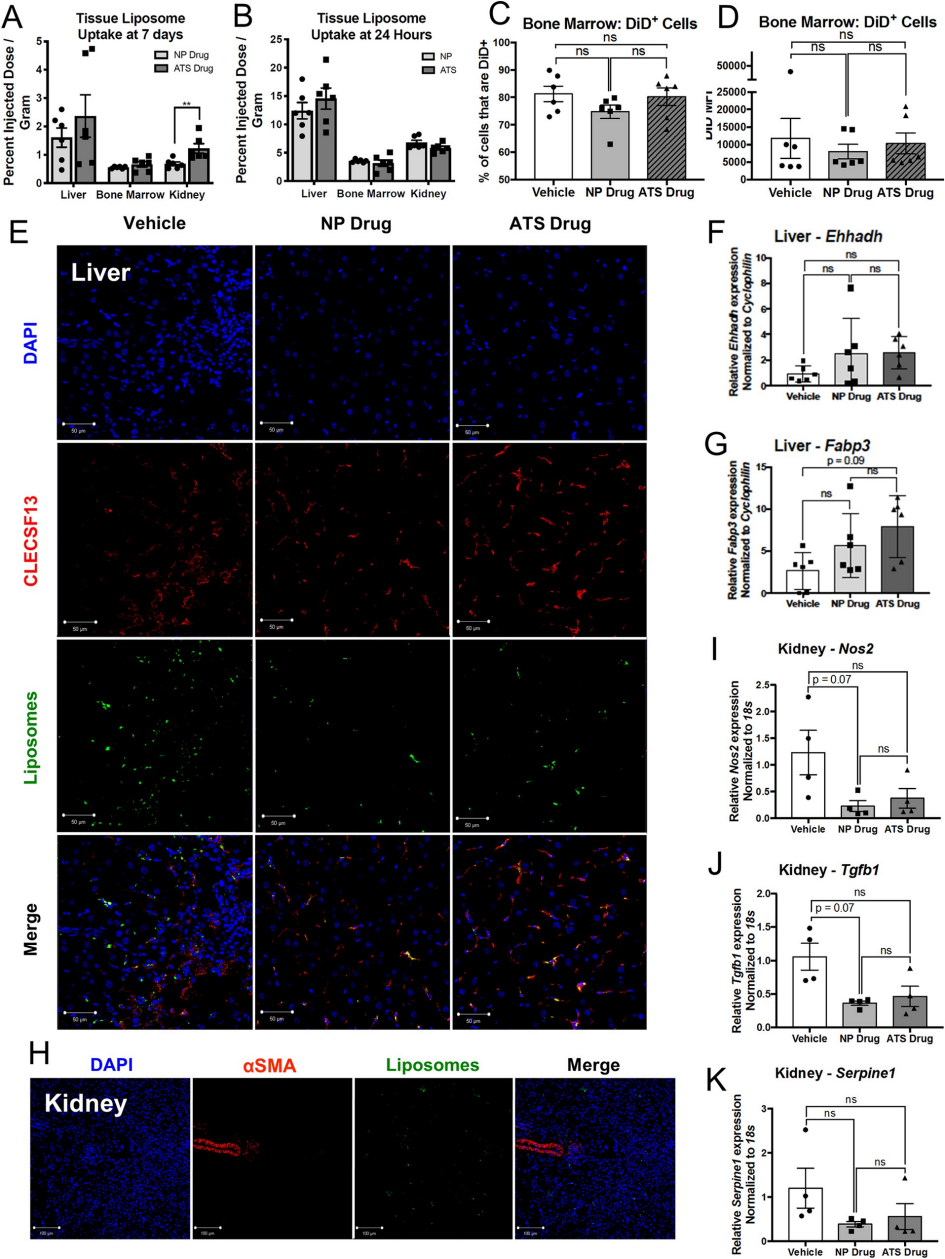
ATS targeting increases uptake in the kidneys and causes no attenuation in uptake in the liver and bone marrow. *Ob/ob* mice used in one-week and one-day studies of liposomal uptake, also had whole tissue *ex vivo* FMT scans performed on their kidneys, bone marrow, and livers (A,B). Bone marrow cells were analyzed by flow cytometry to quantify uptake of DiD-labeled liposomes (C,D). Livers of mice treated with three liposome injections over one week were sectioned and stained for Kupffer cell marker Clecsf13 to visualize cellular uptake of DiD-labeled liposomes (Scale Bar = 50 μm) (E). RNA was harvested from livers of mice treated for one week with untargeted vehicle-, untargeted drug-, or ATS-targeted drug-loaded liposomes to measure mRNA expression levels of *Ehhadh* (F) and *Fabp3* (G). Kidney sections from mice treated for one week with ATS-targeted liposomes were stained with DAPI and αSMA to label nuclei and arteries, respectively (Scale Bar = 100 μm) (H). RNA was harvested from kidneys of mice treated for one week with untargeted vehicle-, untargeted drug-, or ATS-targeted drug-loaded liposomes to measure mRNA expression levels of *Nos2* (I), *Tgfb1* (J), and *Serpine1* (K). Vehicle, vehicle-loaded liposomes; NP Drug, drug-loaded, untargeted liposomes; ATS Drug, drug-loaded, ATS-targeted liposomes. *p<0.05, **p<0.01, Mann-Whitney and Kruskal-Wallis tests.

Examining biodistribution is a necessary step in determining the effects of nanoparticle targeting. Liposome uptake in the liver, bone marrow, and kidneys was studied in *ex vivo* FMT experiments following 24-hour and seven-day treatment with untargeted and ATS-targeted liposomes. In the one-week study, uptake of ATS-targeted liposomes was significantly increased in the kidneys compared to untargeted liposomes, but not in the bone marrow or liver (Fig 4A), but no significant changes were found between the uptake of untargeted and ATS-targeted liposomes at 24 hours in any of the tissues (Fig 4B). However, bone marrow cellular uptake of liposomes was measured by flow cytometry (S3 Fig), and there was no significant change in liposomal uptake observed between treatment groups (Fig 4C and 4D). Immunofluorescence imaging of liver tissue sections revealed that liposomes were associated with Kupffer cells in all liposome treated tissues, with no apparent reduction in liposome uptake observed in the ATS-targeted group relative to untargeted (Fig 4E). Expression of PPAR gene targets in the liver and kidney were measured in response to treatment following the 7-day study. Both liposomal treatment groups had a trending, but not significant increase in expression of PPARα-induced gene targets enoyl-CoA hydratase, 3-hydroxyacyl CoA dehydrogenase (*Ehhadh*) and fatty acid binding protein 3 (*Fabp3*) [38] in the liver relative to the vehicle liposomes, but there was no significant difference in gene expression levels between NP and ATS liposomes (Fig 4F and 4G). Immunofluorescence imaging of kidney sections from each treatment group revealed that liposomes are located perivascularly as well as distal to alpha smooth muscle actin (αSMA)^+^ arteries (Fig 4H and S4 Fig). Treatment with drug-loaded liposomes results in a trend towards a decrease in mRNA expression levels of nitric oxide synthase, inducible (*Nos2*, Fig 4I) and transforming growth factor beta 1 (*Tgfb1*, Fig 4J), but not plasminogen activator inhibitor 1 (*Serpine1*, Fig 4K) in the kidney, all of which have been reported to be PPARγ target genes in the kidney [39, 40]. Despite the increase in uptake of ATS-targeted liposomes in the kidney relative to untargeted liposomes, there was no difference in these PPAR-regulated genes between the ATS and NP liposomes. The unexpected finding of increased kidney uptake of ATS-targeted liposomes highlights the importance of examining the biodistribution during targeting experiments.

## Discussion

In these studies, whole tissue and cellular uptake of ATS-targeted liposomes was quantified to validate selectivity with which these particles were targeted to whole adipose tissue vasculature compared to other cells and tissues that express prohibitin. However, we found that ATS-targeted liposomes did not have enhanced adipose tissue uptake or vasculature-specific targeting, but did see increased uptake in the kidneys. Results from this unbiased, fulsome characterization of DiD-labelled liposome uptake in adipose SVF using flow cytometry demonstrated that ATS-targeting did not enhance liposome uptake in ECs, but did cause a trend towards an increase in uptake in CD45^+^ cells of the subcutaneous adipose SVF compared to untargeted liposomes.

The literature demonstrates that prohibitin is expressed on many different cell types including immune cells, epithelial cells, and endothelial cells [24, 25, 27, 41–44]. With regards to prohibitin expression in endothelial cells *in vivo*, there exists just a few studies [17, 42, 43] demonstrating co-localization of CD31 with prohibitin in a murine aorta [43] and colocalization of lectin with prohibitin in adipose tissue [42]. Kolonin *et al* demonstrate positive prohibitin staining in white adipose tissue localized between adipocytes and in crown-like structures (5). Furthermore, this same study demonstrated the ATS peptide was localized in crown-like structures of white adipose tissue. While the staining may be co-localized with the vasculature, no additional staining for endothelial cell-specific markers was reported nor were the presented images quantified thus creating an important caveat regarding the exact adipose tissue cell types in which prohibitin is expressed and to which ATS localizes.

In our study, immunofluorescence imaging of adipose tissue does indeed show co-localization of CD31 with DiD, showing concordance between our study and the previous literature. However, CD31 staining co-localizes with other non-EC cell types as well as ECs. Non-EC cell types also express CD31, albeit at a lower level of expression [45]. This was particularly apparent in adipose tissue crown-like structures, which are occupied by macrophages as well as B cells and apoptotic adipocytes (S2 Fig) [46]. These data underline the need to perform multi-marker flow cytometry based analysis and include multiple cell markers during immunofluorescence staining to ensure that the correct cell type is being identified when defining liposomal uptake at the cellular level. Since prohibitin is expressed on a multitude of different cell types including CD45^+^ immune cells [24], this suggests that prohibitin expression on adipose ECs may not be high enough relative to other cells within the adipose and other tissues to serve as an effective target for adipose- and EC-selective delivery.

Interesting, we achieved nearly identical results with untargeted liposomes. Nearly all the macrophages within the adipose took up untargeted and ATS-liposomes suggesting that phagocytosis and not targeting was the dominant mechanism of cellular uptake of the liposomal formulations. It is well established that macrophages are an abundant cell type in adipose tissue during obesity and dysmetabolism [47], so their response to PPARα/γ agonism is potentially important. Macrophages have been shown to regulate angiogenesis as well as circulating insulin and glucose levels in animal models of dysmetabolism [48, 49]. While macrophages are known to regulate inflammation in obesity and diabetes and contribute to a pro-inflammatory status [47, 50, 51], they are pushed towards an anti-inflammatory phenotype by PPARα/γ agonism [36]. Thus, macrophage uptake of tesaglitazar-loaded nanoparticles may have a complex role in adipose tissue metabolism and further studies are needed to assess their role in response to PPAR agonists.

Additionally, our flow cytometry data indicates that untargeted and ATS-liposomes were taken up by many other immune and stromal cell types, in addition to macrophages. This included other CD45^+^F4/80^-^ mid- to high side-scatter immune cells, which typically include phagocytes such as dendritic cells; but also B cells and many CD45^-^CD31^-^ stromal cells, which could be fibroblasts, progenitors, and vascular smooth muscle cells. Uptake of liposomes in these cell subsets demonstrates the importance of performing a thorough characterization of liposomal uptake using quantitative methods such as flow cytometry.

We analyzed mice treated with untargeted and targeted liposomes using FMT to determine how ATS targeting changed the biodistribution in the adipose tissue, kidney, liver and bone marrow. We did not find increased uptake of ATS-targeted liposomes in adipose tissue. This is important to note given the well-established benefits of PPAR agonism in adipocytes [52, 53]. Furthermore, liposome uptake as well as drug action, as indicated by *Ehhadh* and *Fabp3* expression, in the liver and bone marrow was unchanged and uptake in the kidneys was increased by ATS targeting relative to untargeted liposomes. The ATS target, prohibitin, is expressed not only on the mitochondrial membrane of podocytes within the kidney, but also in the slit diaphragm (46). The slit diaphragm is a specialized cell junction at the filtration slit localized between podocytes and capillaries. Thus, it is possible that ATS-targeted liposomes may be binding to prohibitin localized at slit diaphragms within the kidney, enhancing uptake in this tissue. Additional studies utilizing the ATS peptide conjugated to KLAKKLAK, a peptide known to cause apoptosis, resulted in decreased adipose tissue mass and weight loss and increased parameters of kidney injury (10). These parameters included increased creatinine levels, increased BUN, and tubular degeneration and single cell necrosis. Ultimately, coupling the findings from these published studies with the lack of significant increases in uptake of ATS-liposomes in adipose tissues and the increase of the liposomal formulations in the kidneys indicates that ATS targeting is not a synergistic strategy for directed delivery of anti-diabetic or–adipogenic compounds to adipose tissue and instead, the drugs benefited from the increase in the numbers of phagocytic cells present in adipose tissue.

ATS-targeted, tesaglitazar-loaded liposomes did not lower fasting insulin or triglyceride levels better than untargeted liposomes. While this was not the hypothesized result based upon a recent study demonstrating that treatment with ATS-targeted nanoparticles loaded with PPARγ agonist rosiglitazone lowered fasting insulin levels over non-targeted controls [20], the decreased efficacy may be due to differences in experimental approach: duration and dosage of the drug treatments. In our study, mice were treated for one week, compared to 25 days in the ATS-targeted nanoparticle rosiglitazone study [20]. While it has been shown that tesaglitazar delivered orally can lower fasting insulin and triglyceride levels after one week of treatment [9], the liposomal delivery of this drug may require a longer time frame to show efficacy due to the altered PK of free drug vs. liposome encapsulated drug. Additionally, the discrepancies may be due to differences in daily dosages. In our study, mice were treated with 400 μg/kg of tesaglitazar per day, which is approximately 40-fold higher than the dose of 10 μg/kg per day given to humans, assuming 1 mg/day for a 90 kg individual. This dosage was shown to lower lipid levels in patients [54]. The 40 mg/kg of rosiglitazone per day given to mice in the rosiglitazone study is approximately 1000-fold higher than the usual dose of 4mg of rosiglitazone for a 90 kg individual. The 25-fold difference between 40-fold tesaglitazar dosage increase and the 1000-fold rosiglitazone dosage increase may play a large role in the discrepancies in the effects observed. This comparison highlights the importance of the physiological dose of a drug when conducting studies assessing its efficacy *in vivo*. Further dose-response studies could determine an appropriate dose of liposomal tesaglitazar that can achieve efficacy without causing unwanted side effects.

In total, our studies utilized FMT, flow cytometry, and microscopy to perform an unbiased, *in vivo* assessment of the capacity of ATS-targeted nanoparticles to target to adipose endothelium, accumulate in adipose tissue, and lower uptake in other tissues such as the kidney, liver, and bone marrow. Given the known efficacy of tesaglitazar in improving metabolic parameters and established role of PPARγ and -α agonists in regulating gene expression in the kidneys and liver, respectively, tesaglitazar served as an excellent candidate compound with which to test targeting efficacy. ATS-targeted delivery of tesaglitazar was expected to increase tesaglitazar accumulation in the adipose tissue while liposomal delivery reduced accumulation in the kidneys by shifting drug clearance to the mononuclear phagocyte system. We found that ATS targeting did not cause significant changes in liposome uptake in adipose tissue, ECs, liver, bone marrow, but did increase uptake in the kidneys. These findings indicate that ATS peptide does not induce the desired biodistribution for targeted delivery of tesaglitazar-loaded liposomes, which had not been shown prior to this study. Targeted delivery is still a worthwhile goal that could be achieved by matching the liposomes with a targeting molecule that provides a more germane biodistribution. New targeting moieties could be selected from the literature, or developed using combinatorial library screens such as phage or yeast display. It is critical that the development of new targeting molecules and targeted nanoparticles include validation experiments examining the biodistribution at the tissue and cellular level. Appropriate evaluation of the biodistribution will improve the efficacy and increase the rate of successful development of targeting therapeutics.

## Supporting information

S1 Fig Characterization of peptide-conjugated liposomes. (A) Nanosight characterization of size distribution of two batches of liposomes used for the 1 week metabolic study. Cryo-TEM images of liposomes were taken before (B) and after remote loading with tesaglitazar (C) to assess the size and structure of the liposomes (Scale Bar = 50 nm). Three batches of liposomes were prepared for the 24h PK study by saline hydration of lipid films followed by extrusion through a 0.2 μm filter. (D) The resulting liposomes size and concentration were characterized by Nanosight.
(TIF)

S2 Fig Lectin and CD68 co-localize in crown-like structures. Whole mounted white adipose tissue (epididymal and subcutaneous) was stained for CD68 and Lectin to identify macrophages and vascular cells. Liposomes were labeled with DiD. Merged images were utilized to identify co-localization of these markers. Yellow boxes mark crown-like structures that are positive for all markers.
(TIF)

S3 Fig Bone marrow and adipose SVF flow cytometry gating strategies. Example flow cytometry gating strategy for identifying DiD+ bone marrow cells (A). Example flow cytometry gating strategy for identifying subsets of CD45^+^hematopoietic cells, CD45^-^ non-hematopoietic cells, CD31^+^ ECs, CD19^+^ B cells, CD3^+^ T cells, and F4/80^+^CD11b^+^ macrophages that are DiD^+^ in adipose SVF (B).
(TIF)

S4 Fig Vehicle and NP liposome localization in the kidney. Kidneys of *ob/ob* mice treated with three injections of vehicle or untargeted liposomes over one week were sectioned and stained for vascular smooth muscle cell marker αSMA to visualize cellular uptake of DiD-labeled liposomes.
(TIF)

S1 File Tissue weights and FMT measurements. (XLSX)

S2 File Insulin, Lipid and Body Weight measurements. (XLSX)
