# Supplementary figures and images for: Importance of thorough tissue and cellular level characterization of targeted drugs in the evaluation of pharmacodynamic effects
Source: PLoS One. 2019 Nov 14;14(11):e0224917. doi: 10.1371/journal.pone.0224917 (PMC6855449; doi:10.1371/journal.pone.0224917)

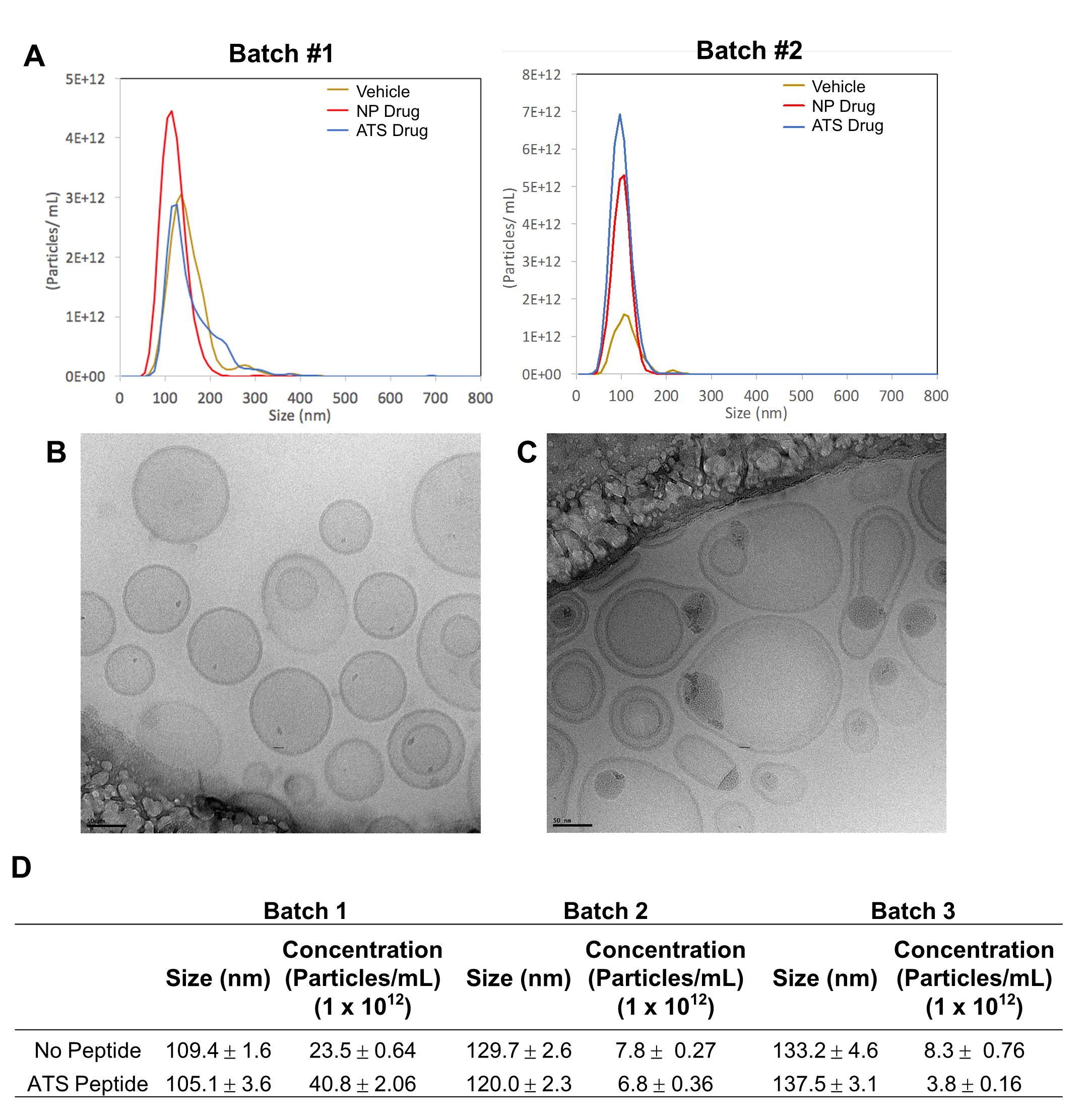

Supplement: S1 Fig — (A) Nanosight characterization of size distribution of two batches of liposomes used for the 1 week metabolic study. Cryo-TEM images of liposomes were taken before (B) and after remote loading with tesaglitazar (C) to assess the size and structure of the liposomes (Scale Bar = 50 nm). Three batches of liposomes were prepared for the 24h PK study by saline hydration of lipid films followed by extrusion through a 0.2 μm filter. (D) The resulting liposomes size and concentration were characterized by Nanosight. (TIF) [file pone.0224917.s001.tif]

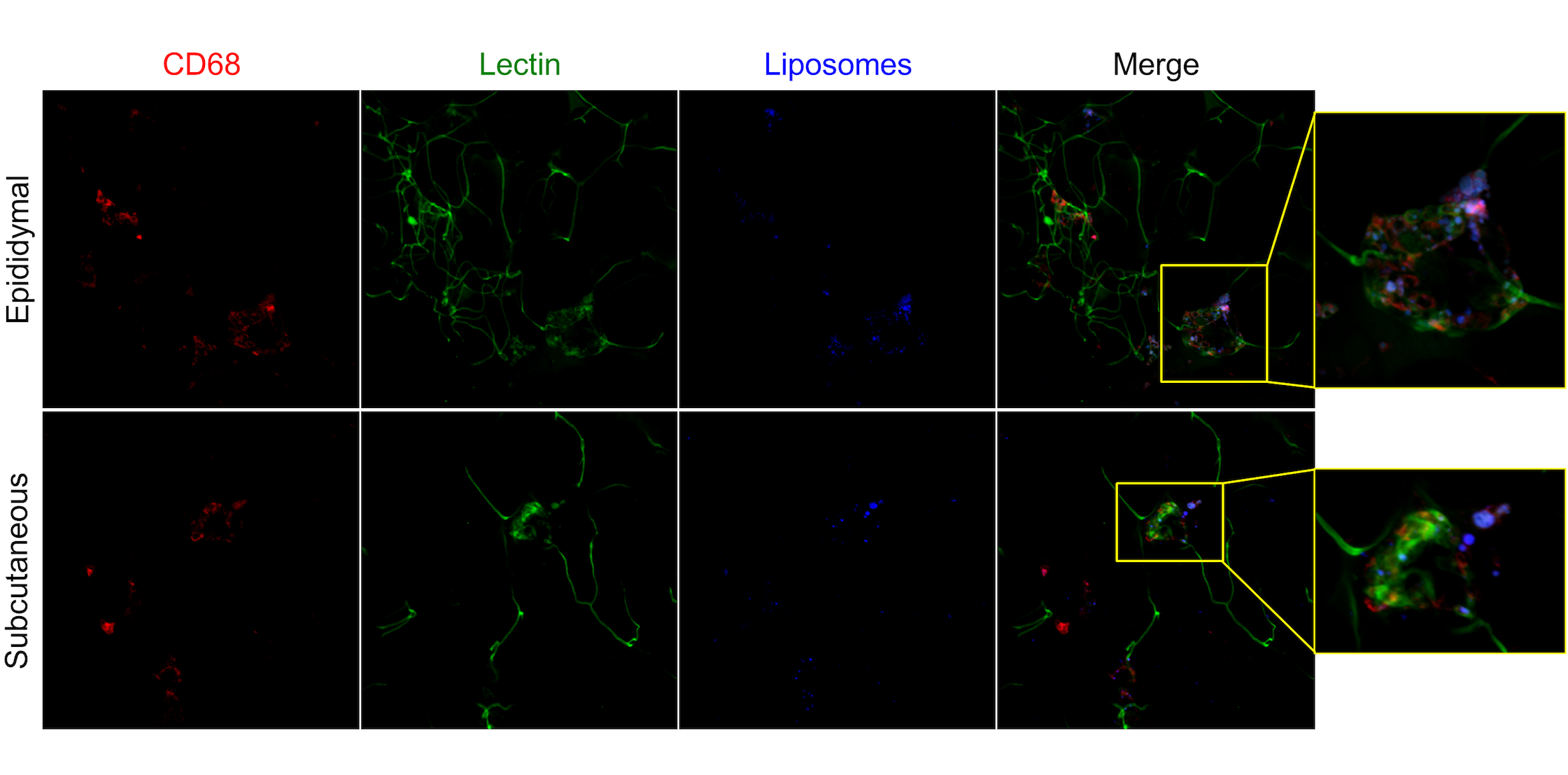

Supplement: S2 Fig — Whole mounted white adipose tissue (epididymal and subcutaneous) was stained for CD68 and Lectin to identify macrophages and vascular cells. Liposomes were labeled with DiD. Merged images were utilized to identify co-localization of these markers. Yellow boxes mark crown-like structures that are positive for all markers. (TIF) [file pone.0224917.s002.tif]

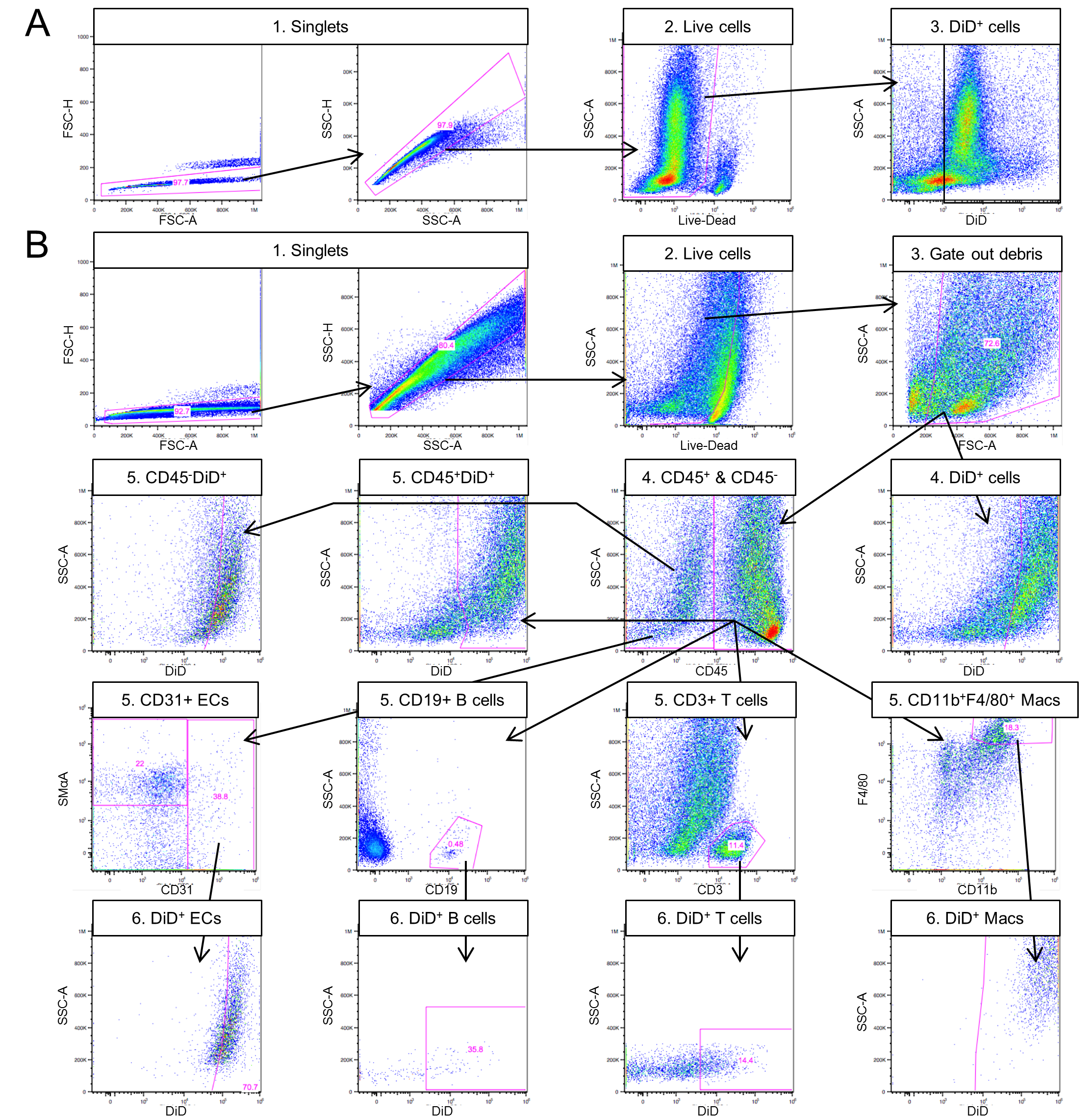

Supplement: S3 Fig — Example flow cytometry gating strategy for identifying DiD+ bone marrow cells (A). Example flow cytometry gating strategy for identifying subsets of CD45+hematopoietic cells, CD45- non-hematopoietic cells, CD31+ ECs, CD19+ B cells, CD3+ T cells, and F4/80+CD11b+ macrophages that are DiD+ in adipose SVF (B). (TIF) [file pone.0224917.s003.tif]

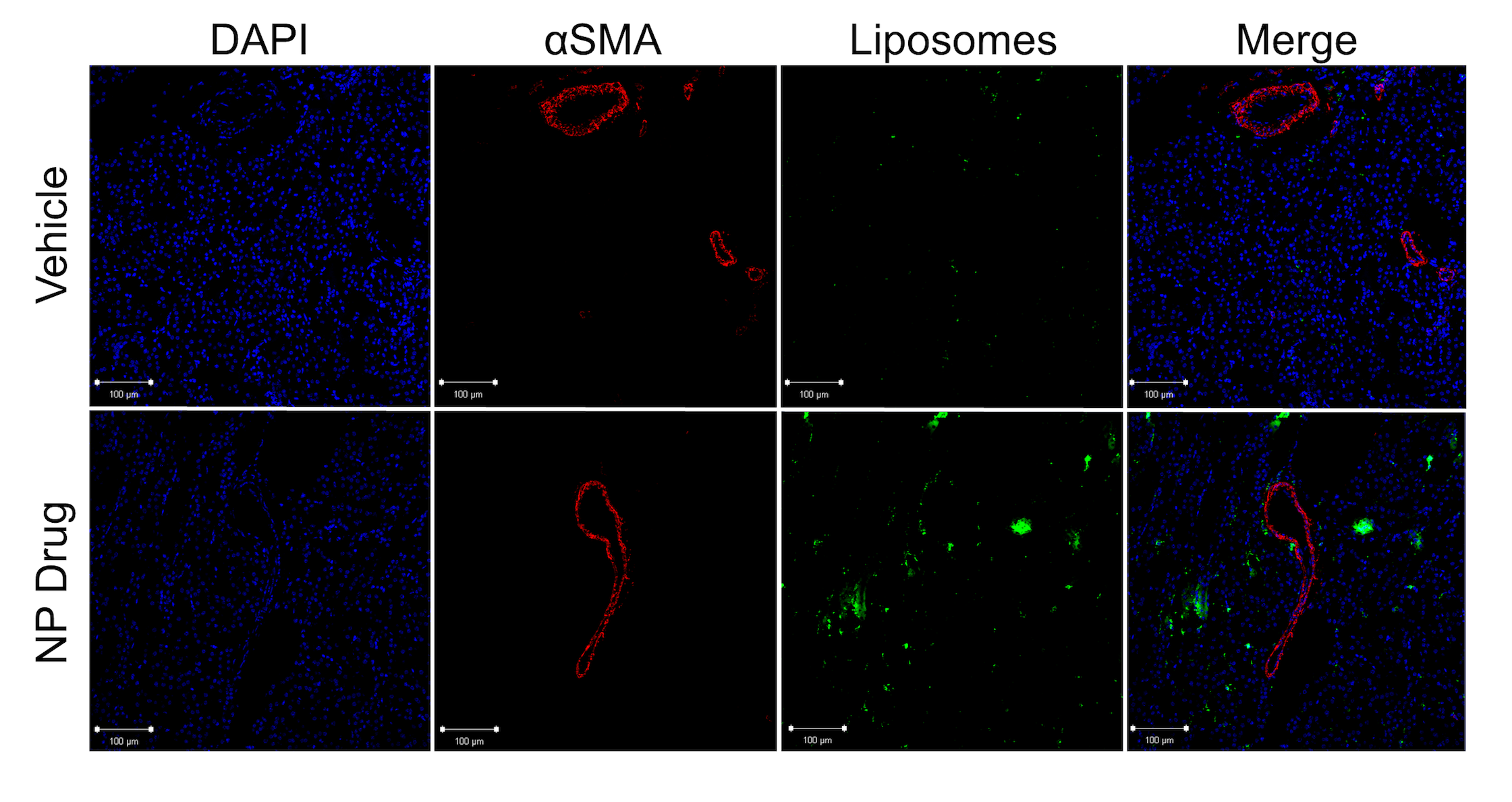

Supplement: S4 Fig — Kidneys of ob/ob mice treated with three injections of vehicle or untargeted liposomes over one week were sectioned and stained for vascular smooth muscle cell marker αSMA to visualize cellular uptake of DiD-labeled liposomes. (TIF) [file pone.0224917.s004.tif]
